# Oxyresveratrol and/or Dapagliflozin Attenuate Doxorubicin-Induced Nephrotoxicity via Modulation of PPAR-γ/Nrf-2/HO-1, NF-κB/TNF-α/Keap-1, and Bcl-2/Caspase-3/ATG-5 signaling pathways in rats

**DOI:** 10.1007/s00210-024-03608-4

**Published:** 2024-12-03

**Authors:** Waleed S M El-Sawy, Marwa M Khalaf, Ali H El-Bahrawy, Basim A S Messiha, Ramadan A M Hemeida

**Affiliations:** 1 Department of Pharmacology & Toxicology, Faculty of Pharmacy, Al-Azhar University, Assiut Branch Assiut, 71524 Egypt; 2 Department of Pharmacology & Toxicology, Faculty of Pharmacy, Beni-Suef University, Beni-Suef, 62514 Egypt; 3 Department of Clinical Pharmacy, Faculty of Pharmacy, Al-Azhar University, Assiut Branch Assiut, 71524 Egypt; 4 Department of Pharmacology & Toxicology, Faculty of Pharmacy, Deraya University, Minya, 61519 Egypt

**Keywords:** Chemotherapy, Doxorubicin, Nephrotoxicity, Oxyresveratrol, Dapagliflozin

## Abstract

**Purpose:**

Among the most undesirable effects that lead to the restriction of doxorubicin (DOX) use in chemotherapy is kidney damage. This research aimed to assess the possible defenses against DOX-induced nephrotoxicity offered by oxyresveratrol (ORES) and/or dapagliflozin (DAPA).

**Methods:**

Five groups of eight male Swiss albino rats each were created from a total of sixty-four. One intravenous injection of DOX (10 mg/kg) was given into the tail vein on the fourteenth day of the experiment; in the meantime, ORES (80 mg/kg) and DAPA (10 mg/kg) were given orally 14 days prior to the DOX injection and 2 days following the DOX injection.

**Results:**

In rats given DOX, ORES and/or DAPA both successfully reduced the kidney weight, kidney/bodyweight ratio, and blood levels of creatinine, uric acid, and urea. They also increased final body weight and albumin serum levels. Additionally, lower serum concentrations of TNF-α and IL-6 were noted, along with a lower kidney content of caspase-3. Furthermore, the expression of the Bcl-2 gene was upregulated, as were the Nrf-2, PPAR-γ, and HO-1 proteins, and there was a downregulation of the ATG-5, Keap-1, and NF-κB renal gene expression. These findings support a decrease in oxidative stress and relief of histopathological alterations.

**Conclusion:**

The current study’s findings suggest that ORES and/or DAPA pretreatment could be a viable therapeutic approach to ameliorate DOX-induced nephrotoxicity.

## Introduction

Anthracyclines, including doxorubicin (DOX), are one of the most effective chemotherapeutics available for various types of cancer. Unluckily, their clinical use is largely restricted due to renal intoxication (Ali et al. 2021). DOX’s capacity to release reactive oxygen species (ROS), which lead to apoptosis, autophagy dysregulation, mitochondrial malfunction, and impaired iron metabolism, could be the cause of DOX-induced nephrotoxicity (Gao et al. 2021).

Different pathways of signaling, such as nuclear factor erythroid-derived 2-like 2 and Kelch-like ECH-associated protein-1 (Nrf-2/Keap-1), that impact the cytotoxicity observed in the affected cells, can be triggered by oxidative stress (Yu and Xiao 2021). The negative regulator Keap-1 suppresses Nrf-2-dependent transcription during homeostasis. But in response to oxidative damage, Nrf-2 migrates towards the nucleus, leaves Keap-1, and attaches itself to the electrophile response elements (ERE) of multiple cell-protective genes, suppressing the nuclear factor Kappa-B (NF-κB) and releasing many antioxidant enzymes, including heme oxygenase-1 (HO-1), which in turn suppresses tumor necrosis factor-alpha (TNF-α) and interleukin-6 (IL-6) (Turpaev 2013; Younis and Ghanim 2022).

Moreover, peroxisome cellular proliferator-activated receptor-γ (PPAR-γ) serves as a nutritional sensor that controls several homeostatic activities, such as the metabolism of glucose and fat (Aboonabi and Aboonabi 2020). It is thought that in the renal system, PPAR-γ regulates oxidative stress, the antioxidant response, and inflammatory illnesses (Ma et al. 2020).

Conversely, DOX can induce pro-apoptotic proteases such as caspase-3, which in turn can start the kidney’s apoptotic pathway (Xiang et al. 2021). The molecule called B-cell lymphoma-2 (Bcl-2) has anti-apoptotic properties that inhibit caspase-3’s function. Bcl-2 activation suggests a decrease in renal cell apoptosis (Kubat et al. 2021). Moreover, the physiological catabolic process, autophagy, is crucial for cell survival because it scavenges damaged or aging cellular macromolecules and combats cellular stress (Jung et al. 2020). It’s known that ROS propagation is associated with autophagy transcription genes such as autophagy-related protein-5 (ATG-5) (Yun et al. 2020).

Oxyresveratrol (ORES), also referred to as 2,3′,4,5′-tetrahydroxystilbene, is a hydroxyl-substituted stilbene. An ethylene bridge unites the two aromatic rings that make up these monomeric stilbenes, and ORES, a naturally occurring stilbene, belongs to the hydroxystilbenoids phytochemical group. Its molecular structure is similar to that of the well-known phytochemical resveratrol (3,4′,5-trihydroxy stilbene), but ORES has a hydroxyl group on carbon 2 (Likhitwitayawuid 2021). Many plants, including those in the Liliaceae and Moraceae families, have ORES in their fruit, stem, roots, and leaves (Ti et al. 2011). Strong anti-inflammatory, anticancer, antiviral, antibacterial, hypoglycemic, and neuroprotective properties are possessed by ORES (Xu et al. 2014).

Dapagliflozin (DAPA) is the first drug to treat type 2 diabetes in a new class of antidiabetic medications. It is a member of the sodium-glucose co-transporter-2 (SGLT-2) inhibitor class (Scheen 2015). Recent studies have shown that DAPA significantly lowers oxidative stress and inflammatory indicators (Abdel-Wahab et al. 2018).

The antioxidant vitamin E (Vit. E), also known as alpha-tocopherol, is soluble in lipids and defends biological cellular membranes against lipid peroxidation. Previous research has shown that Vit. E has great efficacy in fighting inflammation, oxidative damage, and apoptosis (Singh et al. 2005; Kurutas 2015).

This study offers an additional explanation of the various detrimental routes through which DOX causes acute nephrotoxicity in experimental animals, specifically rats. These pathways consist of autophagy, apoptosis, inflammation, and oxidative stress. A variety of techniques, including spectrophotometers, ELISA, qRT-PCR, immunohistochemistry, histopathology, and Western blotting, were used to measure a number of parameters and proteins through these pathways. Also, the current research sets out to assess the roles that various signaling pathways, including Keap-1/Nrf-2/HO-1, TNF-α/NF-κB/PPAR-γ, and ATG-5/Bcl-2/Caspase-3, play in the development and prevention of kidney injury. It also sought to determine whether ORES and/or DAPA could provide any defenses against renal injury caused by DOX.

## Materials and methods

### Chemicals and medications

Adriamycin (doxorubicin HCl) injectable solution was obtained from Hikma Specialized Pharmaceuticals Co., Egypt. ORES was obtained from Shaanxi Yi An Biological Technology Co., Ltd., China. DAPA was obtained as a kind gift from AstraZeneca Saudi Arabia Co., for Pharmaceutical and Biopharmaceutical Industries., Kingdom of Saudi Arabia. Vit. E was obtained from Pharco Pharmaceuticals Co., Egypt. Biodiagnostic Company (Cairo, Egypt) provided the assay kits for albumin (AB 10 10), creatinine (CR 12 50), urea (UR 21 10), uric acid (UA 21 20), and total antioxidant capacity (TAC) (TA 25 13). The ELISA kits for TNF-α (Cat: KBT-K0331196), caspase-3 (Cat: CSB-E08857r), and IL-6 (Cat: E-EL-R0015) were obtained from Komabiotech Inc. (Seoul, Korea), Cusabio^®^Biotech (Wuhan, China), and Elabscience Biotech Co. (USA), respectively. The remaining chemicals had consistent analytical values and were obtained from different sources.

### Experimental animals

From the main animal house, sixty-four mature male Swiss albino rats (10 weeks, 210 ± 10 g) were obtained (Faculty of Medicine, Assiut University, Egypt). Eight caged groups of animals were fed commercial pellet food on a regular basis and given unlimited access to water. A 12-hour light-dark cycle was used to maintain the temperature at 25 ± 2 °C. The animals received a two-week period of adaptation prior to investigations in the boys’ pharmacy research lab at Al-Azhar University in Assiut, Egypt.

## Experimental design

The animals were weighed and then split up into five groups, each with eight rats:

### Group I

Only the vehicle was received by rats. The typical (negative) control group was this one.

### Group II

Rats were received a single intravenous dose of DOX (10 mg/kg) via the tail vein on the fourteenth day of the study (El-Sayed et al. 2016).

### Group III

A dosage of ORES was given orally to rats (80 mg/kg) for a continuous 14-day period (Jia et al. 2018). On day 14, the rats were administered one intravenous dose of DOX (10 mg/kg). Two days later, they received an oral dose of ORES (80 mg/kg).

### Group IV

For 14 days in a row, rats received 10 mg/kg orally of DAPA (Chang et al. 2016). They were given one intravenous dose of DOX (10 mg/kg) on day 14, and they were given another daily dose of 10 mg/kg orally of DAPA for the next two days.

### Group V

Rats received for 14 days a daily dose of Vit. E at a rate of 1 g/kg orally in a row as part of a pretreatment (Nematbakhsh et al. 2012). On day 14, the rats were given one intravenous dose of DOX (10 mg/kg), after which they continued receiving Vit. E at a daily rate for two days in a row.

The drug solutions were freshly prepared prior to administration. In the control group, each 1 kg of animals received 0.5 ml of dimethyl sulfoxide (DMSO) (0.5%) diluted with 4.5 ml normal saline (5 ml/kg/day p.o.). The selected doses of the drugs in the current investigation, including ORES, DAPA, and Vit. E, were each dissolved in 0.5 ml of DMSO (0.5%) diluted with 4.5 ml of normal saline (5 ml/kg/day p.o.).

An experiment was done to explain the effect of test agents alone in order to assure their safety before looking at how test agents affected the toxicity of DOX.

### Preparing tissues and serum

Rats were anesthetized with light ether on the seventeenth day of the experiment, and using a non-heparinized tube, blood samples were extracted from the retro-orbital venous plexus. At 4000 rpm and at 4 ºC, blood samples were centrifuged for 10 min in order to isolate serum. Later, it was kept at -20 ºC until renal function parameters, TAC, IL-6, and TNF-α, were biochemically analyzed. After opening the abdomen, the kidneys were taken out, cleaned three times in ice-cold saline from fat and tissue debris, and dried between two sheets of filter paper. For histological analysis, one kidney was submerged in a 10% buffered formalin solution. After cutting the second kidney in half, the first half was homogenized using phosphate buffer in an ice-cold saline solution (with a pH of 7.0) to create a 10% homogenate. After centrifugation for 15 min at -4 ºC and 4000 rpm, the kidney contents of the homogenate were measured for caspase-3, malondialdehyde (MDA), nitrite (NO_2_^-^), reduced glutathione (GSH), and superoxide dismutase (SOD) activity. Subsequently, -80 ºC was maintained for the supernatant. The gene expression of Keap-1, ATG-5, Bcl-2, and NF-κB was measured in the second half by utilizing the quantitative real-time polymerase chain reaction (qRT-PCR) method after being submerged in liquid nitrogen at a temperature of -80 ºC. Additionally, assessment of Nrf-2 and PPAR-γ protein expression using Western blot analysis and determination of HO-1 expression by immunohistochemical staining.

### Examination of the kidney-to-body weight ratio and body weight

At the start (starting body weight) and finish (ultimate body weight) of the study, the total body weight for all five groups was noted. where (final weight - beginning weight)/final weight x 100 is used to get the percentage change in total body weight. The weights of the two kidneys were divided by the total body weight at the end and multiplied by 100 to determine the kidney-to-body weight ratio.

### Biochemical examination

The serum was used for the calorimetric measurements of creatinine, albumin, urea, and uric acid using standard creatinine solution (2 mg/dL), standard albumin (4 g/dL), standard urea (50 mg/dL), and standard uric acid (6 mg/dL), following along with the procedures of Narimani et al. (2020), Yoshihiro et al. (2020), Pundir et al. (2019), and Ryan et al. (2019), respectively. The Aydin (2015) protocol was followed to measure the serum levels of IL-6 and TNF-α as well as the concentration of caspase-3 in the kidney tissue. A standard curve made up of multiple serial standard dilutions was used to calculate concentrations.

### Evaluation of oxidative stress parameters

The tissue homogenate was assessed using the techniques outlined by Tsikas (2017**)** for the kidney contents of MDA determination. As one molecule of MDA reacts with two molecules of thiobarbituric acid at a low pH, the resulting pink color is measured by a spectrophotometer and used to determine the amount of thiobarbituric acid reactive substances. The Tsikas (2007**)** technique was used for renal NO_2_^-^ content assessment. Based on the presence of nitrite in an acidic medium, the formed nitrous acid diazotizes sulphanilamide, and the product is coupled with N-(1-naphthyl) ethylenediamine dihydrochloride. The resulting azo dye has a bright reddish-purple color, which can be measured colorimetrically. The Shcherbatykh et al. (2022) methodology was used for the determination of renal GSH contents. The assay is based on the reduction of 5,5′ dithio-bis [2-nitrobenzoic acid] by the SH-group (mainly GSH) present in the test sample to form one molecule of 2-nitro-5-mercaptobenzoic acid with an intense, stable yellow color that can be measured spectrophotometrically. While the method described by Kumar et al. (2020) is used for the assessment of SOD activity in the kidney. This kinetic assay is based on the inhibition of pyrogallol auto-oxidation by SOD, and the inhibition is directly proportional to the activity of SOD in the tested sample. Moreover, TAC in serum was detected using the Koracevic et al. (2001) approach. The reaction of the antioxidants in the sample with a defined amount of exogenously provided hydrogen peroxide. The residual hydrogen peroxide is determined colorimetrically by an enzymatic reaction that involves the conversion of 3,5-dichloro-2-hydroxybenzensulphonate to a colored product.

### Renal gene expression analysis

ReverTra Ace (Toyobo Co., Ltd., Osaka, Japan) and Arbitrary Primer (Thermo Fisher Scientific, Inc., Waltham, MA, USA) were used for first-strand cDNA synthesis after total kidney RNA was extracted using the Sepasol reagent (Nacalai Tesque, Inc., Kyoto, Japan). To perform the qRT-PCR procedure, Thermo Fisher Scientific, Inc., Waltham, MA, USA, provided the StepOnePlus RT-PCR Kit with Fast SYBR Green Master Mix Reagent. NF-κB, ATG-5, Bcl-2, Keap1, and β-actin (the endogenous reference gene) were the primers selected for RT-PCR (Table 1). The specially made primers that were acquired were from Thermo Fisher Scientific, Inc., located in Waltham, MA, USA. The comparative CT method was used to ascertain the relative gene expression, according to Livak and Schmittgen (2001).

**Table 1:** Primer sequences for qRT-PCR

Target gene	Forward	Reverse
ATG-5	5^−^ATGACTAGCCGGGGGAACACC 3^−^	3^−^CCAGTTTACCATCACTGCC 5^−^
Bcl-2	5^−^ CTCAGCCAGCCAGTGACATA 3^−^	3^−^CCGTGATCCAGATACAT 5^−^
Keap-1	5^−^GGACGGCAACACTGATTC 3^−^	3^−^TCGTCTCGATCTGGCTCATA 5^−^
NF-κB	5^−^TGGGACGACACCTCTACACA 3^−^	3^−^GGAGCTCATCTCATAGTTGTCC 5^−^
β-actin	5^−^CTCCTGCTTGCTGATCCACATC 3^−^	3^−^GATTACTGCCCTGGCTCCTAGC 5^−^

### Renal protein expression assessment

The protocols described by Hnasko and Hnasko (2015) were followed while using the Western blotting technique. Protein samples (25 µg) were gently blended, boiled for 5 min in loading buffer (sodium dodecyl sulphate (SDS)), left for 5 min on ice, and arranged on a polyacrylamide-SDS gel. After the samples were separated using a gel electrophoresis device (Cleaver, UK), they were run via a Bio-Rad semi-dry electroblotter (USA) for 30 min on polyvinylidene fluoride membranes. To reduce the indefinite antibody/membrane protein interaction, the membrane was coated with a mixture of non-fat dried milk (5%) in Tween 20-Tris-buffered saline (T-TBS) for two hours at 37 ºC. Then, the membrane was left at 4 ºC for 12 h with a particular dilution of each primary antibody. We employed the Elabscience Biotech Co. (USA) primary polyclonal rabbit antibodies we had chosen to target the proteins β-actin (Cat: E-AB-20058), PPAR-γ (Cat: E-AB-32647), and Nrf-2 (Cat: E-AB-32280). Following that, T-TBS was used to wash the blots three times for 10 min each. Subsequently, the suitable horseradish peroxidase (HRP)-linked secondary antibody (Dako) was then incubated on the membrane at room temperature for an extra hour. Three T-TBS washings, each lasting 10 min, came next. The blot was exposed to the chemi-luminescent Western ECL substrate (PerkinElmer, Waltham, MA). A ChemiDoc imager (BioRad, USA) was used to record the chemi-luminescent signals. Following measurement, β-actin was used to modify the band intensities.

### Histopathological evaluation of the kidney

The renal specimens were stored in neutral buffered formalin (10%) for a duration of 72 h. After being washed in Xylene and treated with progressively higher grades of ethyl alcohol, specimens were invaded and inserted into tissue embedding media (paraplast) (Leica Biosystems). Sections 5µ thick were cut with a rotatory microtome to show the tissue parenchyma in various samples, following which they were put on glass slides. The standard staining technique for tissue slices intended for light microscopic examination was hematoxylin (H) and eosin (E). The tissue sections (at least 5–10 sections) were viewed in a blind fashion by an experienced histologist. Culling (2013) lists all accepted practices and guidelines for sample fixing and staining.

### Renal immunohistochemical staining

Using 4 μm-thick paraffin, kidney tissue slices were created following the manufacturer’s instructions. Sections of recovered deparaffinized tissue were incubated with anti-HO-1 (MA1-112; Thermo Fisher, 1:100) for an overnight period at 4 °C after being exposed to 0.3% H_2_O_2_ for 20 min. Following a PBS rinse, the tissue slices were subjected to a 20-minute treatment using the DAKO HRP-secondary antibody Envision kit. Following this, they underwent another washing and were incubated in diaminobenzidine (DAB) for a further 15 min. Before being covered with a slide for microscopic inspection, PBS washing, hematoxylin counterstaining, xylene cleaning, and dehydration are required (Ramos-Vara and Miller 2014). Furthermore, six non-overlapping areas (400X) were chosen at random from each sample’s renal tissue and scanned in order to calculate the area percentage of HO-1’s immunohistochemistry expression levels in sections that had been stained. The histological analysis was done by using a Full-HD microscope (German manufacturer Leica Microsystems GmbH) and calculating the mean and SEM for all groups to determine the mean area of each slide.

### Analytical statistics

The results are shown as means ± SEM. Following a one-way ANOVA analysis of the data, the Tukey-Kramer multiple comparisons test was performed on each of the five groups to look for statistically significant group differences. P-values less than 0.05 were taken into account in every comparison. All statistical analysis was performed using GraphPad Prism version 8.0 for Windows (GraphPad Software, San Diego, California, USA).

## Results

### Impacts of ORES, DAPA, and Vit. E pretreatment on the kidney weight, kidney-to-body weight ratio, and ultimate body weight

**Fig. 1:**
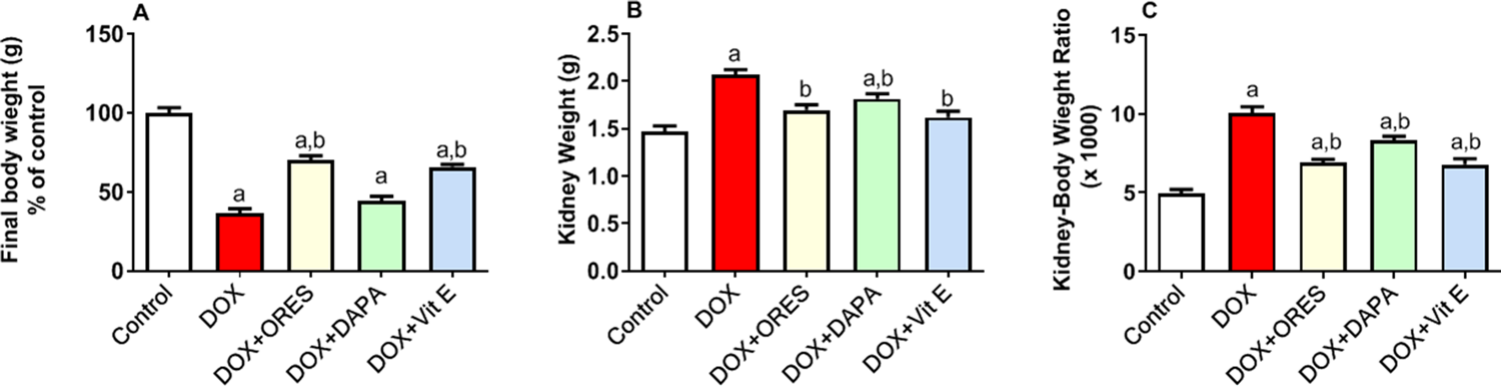
Effects of ORES, DAPA, and Vit. E administration on kidney weight (**B**), kidney-to body weight ratio (**C**), and final body weight (**A**) in rats given DOX The data were displayed as means ± SEM. Using one-way ANOVA and Tukey's test for multiple comparisons across groups at *p* < 0.05, the results were shown to be: ^a^ Significantly different from the control saline group; ^b^ Significantly different from the DOX-administered group.

When compared to the normal control group, the injection of DOX obviously causes a significant decline in the final body weight by 0.3 times as well as an increase in the kidney weight and kidney-to-body-weight ratio by 0.41 and 10.3 times, respectively, as Fig. 1(A-C) illustrates. In comparison to the latter group, rats treated with ORES, DAPA, and Vit. E prior to DOX therapy showed a significant decrease in kidney weight by 0.18, 0.12, and 0.22 times, respectively, and kidney-to-body weight ratio by 0.31, 0.17, and 0.33 times, respectively. Moreover, the ORES and Vit. E groups showed increases in final body weight by 0.18 and 0.16 times, respectively, in contrast to the DOX-treated rats; in contrast, the DAPA-treated rats showed no discernible changes.

### Effect of ORES, DAPA, and Vit. E on renal function parameters

When compared to the controls, the single-dose injection of DOX resulted in considerably higher levels of creatinine, urea, and uric acid by 3.61, 1.01, and 1.31 times, respectively, and significantly lower levels of albumin by 0.68 times **(**Table 2**)**. On the other hand, serum albumin levels were significantly greater in the ORES, DAPA, and Vit. E-treated group than in the DOX-treated group by 1.56, 0.87, and 1.69 times, respectively, while creatinine, urea, and uric acid levels were significantly lower by 0.46, 0.4, and 0.53 times, respectively, and 0.31, 0.29, and 0.29 times, respectively, and 0.38, 0.24, and 0.45 times, respectively. These results imply that the use of these drugs resulted in the restoration of renal function.

**Table 2:** Impact of ORES, DAPA, and Vit. E administration on serum levels of kidney function markers (albumin, creatinine, uric acid, and urea) in rats given DOX

Parameters Groups	Urea (mg/dl)	Uric acid (mg/dl)	Creatinine (mg/dl)	Albumin (g/dl)
**Control**	32.55 ± 1.951	2.10 ± 0.080	0.56 ± 0.036	5.65 ± 0.128
**DOX**	65.47 ± 3.952^a^	4.86 ± 0.156^a^	2.58 ± 0.076^a^	1.80 ± 0.148^a^
**DOX + ORES**	45.29 ± 1.363^a, b^	3.02 ± 0.123^a, b^	1.39 ± 0.071^a, b^	4.61 ± 0.143^a, b^
**DOX + DAPA**	46.41 ± 1.648^a, b^	3.71 ± 0.172^a, b^	1.56 ± 0.069^a, b^	3.37 ± 0.172^a, b^
**DOX + Vit. E**	46.29 ± 1.733^a, b^	2.66 ± 0.160^b^	1.21 ± 0.084^a, b^	4.84 ± 0.072^a, b^

The data were displayed as means ± SEM. Using one-way ANOVA and Tukey’s test for multiple comparisons across groups at *p* < 0.05, the results were shown to be: ^a^ Significantly different from the control saline group; ^b^ Significantly different from the DOX-administered group

### Treatment with ORES, DAPA, and Vit. E affects oxidative stress indicators

In comparison with the healthy control group, our study revealed that the kidney tissues in this investigation showed a considerable rise in MDA and NO_2_^-^ by 9.33 and 2.91 times, respectively, following the administration of DOX. Furthermore, there was a rapid decrease in serum TAC by 0.51 times, tissue contents of GSH by 0.7 times, and SOD enzymatic activity by 0.67 times, which suggested an increase in markers associated with oxidative stress. In contrast, the kidney contents of GSH and SOD, as well as the serum level of TAC, were significantly elevated by 1.51, 1.37, and 1.64 times, respectively, and 1.93, 0.9, and 1.55 times, respectively, and 0.57, 0.22, and 0.79 times, respectively, and the tissue contents of MDA and NO_2_^-^ were significantly decreased by 0.65, 0.36, and 0.61 times, respectively, and 0.65, 0.3, and 0.63 times, respectively, when the rats received ORES, DAPA, and Vit. E prior to treatment **(**Table 3**)**.

**Table 3:** Effect of ORES, DAPA, and Vit. E pretreatment on oxidative stress indicators in rats receiving DOX

Parameters Groups	MDA (nmol/g tissue)	NO_2_^−^ (µmol/g tissue)	GSH (µmol/g tissue)	SOD (U/g tissue)	TAC (mmol/L)
**Control**	31.4 ± 2.54	0.82 ± 0.028	11.72 ± 0.207	421.7 ± 5.86	1.32 ± 0.025
**DOX**	324.4 ± 13.83^a^	3.21 ± 0.026^a^	3.50 ± 0.197^a^	138.1 ± 11.63^a^	0.65 ± 0.024^a^
**DOX + ORES**	115.3 ± 3.86^a, b^	1.12 ± 0.022^a, b^	8.79 ± 0.168^a, b^	404.1 ± 3.82^b^	1.02 ± 0.021^a, b^
**DOX + DAPA**	207.5 ± 4.43^a, b^	2.25 ± 0.057^a, b^	8.30 ± 0.226^a, b^	262 ± 19.69^a, b^	0.79 ± 0.018^a, b^
**DOX + Vit. E**	127.6 ± 2.42^a, b^	1.18 ± 0.016^a, b^	9.25 ± 0.222^a, b^	352.5 ± 8.41^a, b^	1.16 ± 0.016^a, b^

The data were displayed as means ± SEM. Using one-way ANOVA and Tukey’s test for multiple comparisons across groups at *p* < 0.05, the results were shown to be: ^a^ Significantly different from the control saline group; ^b^ Significantly different from the DOX-administered group

### The impacts of ORES, DAPA, and Vit. E on apoptotic and inflammatory biomarkers

Our results **(**Table 4**)** using the ELISA technique demonstrated that DOX therapy significantly enhanced the apoptotic and inflammatory markers, such as caspase-3 by 5.88 times, TNF-α by 1.99 times, and IL-6 by 3.83 times, compared to the baseline control group. On the other hand, compared to the DOX group, the co-administration of ORES, DAPA, and Vit. E resulted in a noteworthy reduction in blood levels of TNF-α and IL-6 by 0.64, 0.28, and 0.5 times, respectively, and 0.37, 0.21, and 0.28 times, respectively, along with a notable decrease in the quantity of caspase-3 by 0.54, 0.2, and 0.63 times, respectively, in the kidney.

**Table 4:** Effect of ORES, DAPA, and Vit. E treatment on apoptotic and inflammatory indicators in rats administered DOX

Parameters Groups	TNF-α (pg/ml)	IL-6 (pg/ml)	Caspase-3 (ng/g tissue)
**Control**	28.15 ± 2.21	27.14 ± 2.08	3.13 ± 0.25
**DOX**	84.20 ± 2.33^a^	131 ± 3.06^a^	21.54 ± 0.91^a^
**DOX + ORES**	45.21 ± 2.12^a, b^	83.18 ± 2.94^a, b^	9.98 ± 0.72^a, b^
**DOX + DAPA**	60.26 ± 2.03^a, b^	104 ± 2.21^a, b^	17.15 ± 0.88^a.b^
**DOX + Vit. E**	42.08 ± 2.60^a, b^	93.97 ± 2.94^a, b^	7.99 ± 0.35^a, b^

The data were displayed as means ± SEM. Using one-way ANOVA and Tukey’s test for multiple comparisons across groups at *p* < 0.05, the results were shown to be: ^a^ Significantly different from the control saline group; ^b^ Significantly different from the DOX-administered group

### Treatment with ORES, DAPA, and Vit. E impacts the renal gene expression levels

**Fig. 2:**
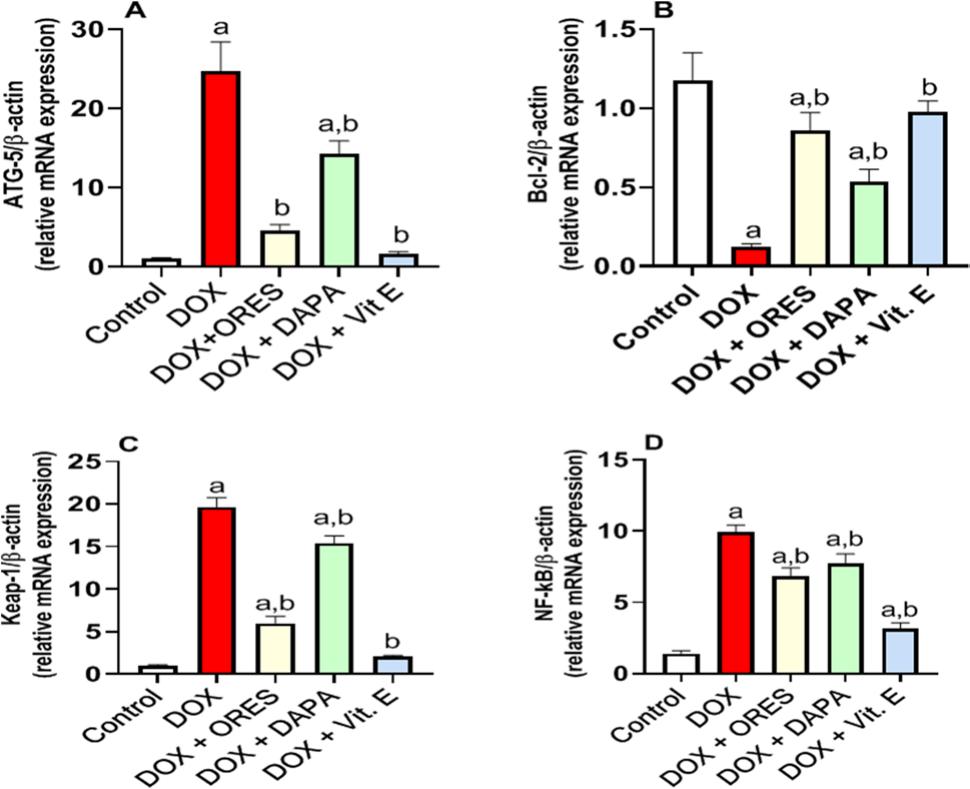
Impact of ORES, DAPA, and Vit. E therapy on renal gene expression levels in rats administered DOX A representative graph of gene expression level analysis shows ATG-5 mRNA (**A**), Bcl-2 mRNA (**B**), Keap-1 mRNA (**C**), and NF-kB mRNA (**D**). The data were displayed as means ± SEM. Using one-way ANOVA and Tukey's test for multiple comparisons across groups at *p* < 0.05, the results were shown to be:^a^ Significantly different from the control saline group; ^b^ Significantly different from the DOX-administered group

Assessment of mRNA expression in the kidney by applying the qRT-PCR technique indicated that DOX injection considerably raised ATG-5, Keap-1, and NF-κB renal gene expression by 23, 19, and 6-fold, respectively, and dramatically lowered Bcl-2 gene expression by −0.9-fold compared to the normal group. Alternatively, concurrent treatment of animals with ORES, DAPA, and Vit. E markedly ameliorated these molecular alterations, as demonstrated by a large drop in ATG-5 by −0.8, −0.4, and − 0.9-fold respectively, Keap-1 by −0.7, −0.2, and − 0.9-fold respectively, and NF-κB gene expression by −0.3, −0.2, and − 0.7-fold respectively, and a considerable rise in Bcl-2 expression by 6, 3, and 7-fold, respectively, against the DOX-treated group **(**Fig. 2(A-D)).

### The effect of ORES, DAPA, and Vit. E therapy on renal protein expression levels

**Fig. 3:**
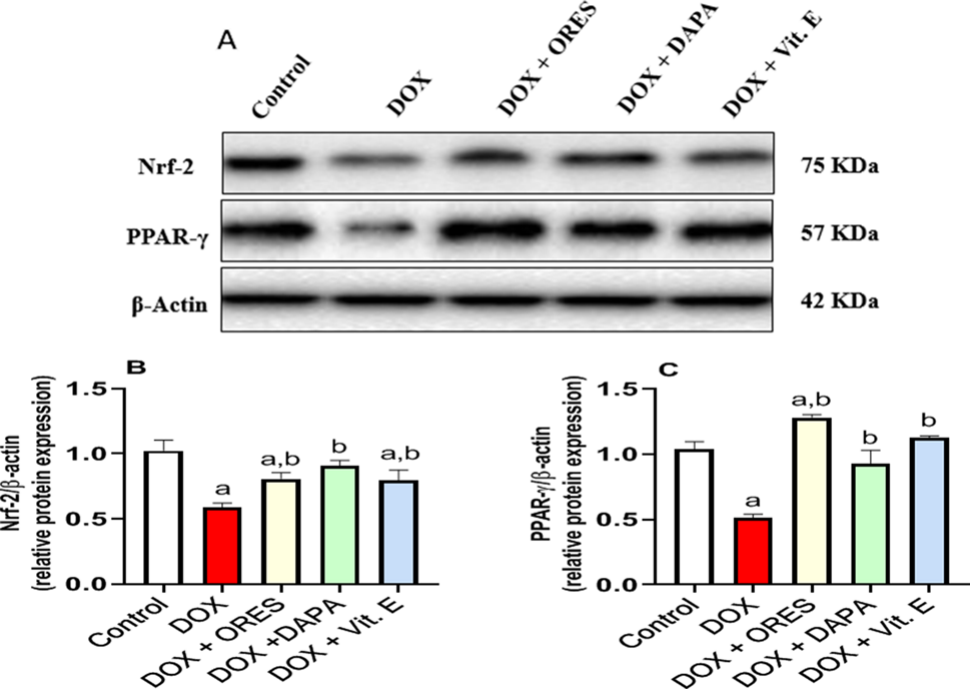
Effect of ORES, DAPA, and Vit. E treatment on renal protein expression levels in DOX-administered rats The upper two panels display the level of Nrf-2 and PPAR-γ protein expression statistical analysis (**B** and **C**), respectively, while the upper panel displays the representative gel of the corresponding protein analysis normalized to β-actin (**A**). The data were displayed as means ± SEM. Using one-way ANOVA and Tukey's test for multiple comparisons across groups at *p *< 0.05, the results were shown to be: ^a^ Significantly different from the control saline group; ^b ^Significantly different from the DOX-administered group

This investigation used Western blotting to evaluate the impact of DOX treatment on renal Nrf-2 and PPAR-γ protein expression, as shown in Fig. 3(A-C). Our findings demonstrated that the rats treated with DOX had significantly lower levels of Nrf-2 and PPAR-γ protein expression by −0.4 and − 0.5-fold, respectively. Nonetheless, the rats treated with ORES, DAPA, and Vit. E exhibited notably higher Nrf-2 and PPAR-γ expression levels by 0.4, 0.6, and 0.4-fold, respectively, and 1.5, 0.8, and 1.2-fold, respectively, than the DOX-treated group.

### Treatment with ORES, DAPA, and Vit. E ameliorates DOX-induced renal histopathological alterations

**Fig. 4:**
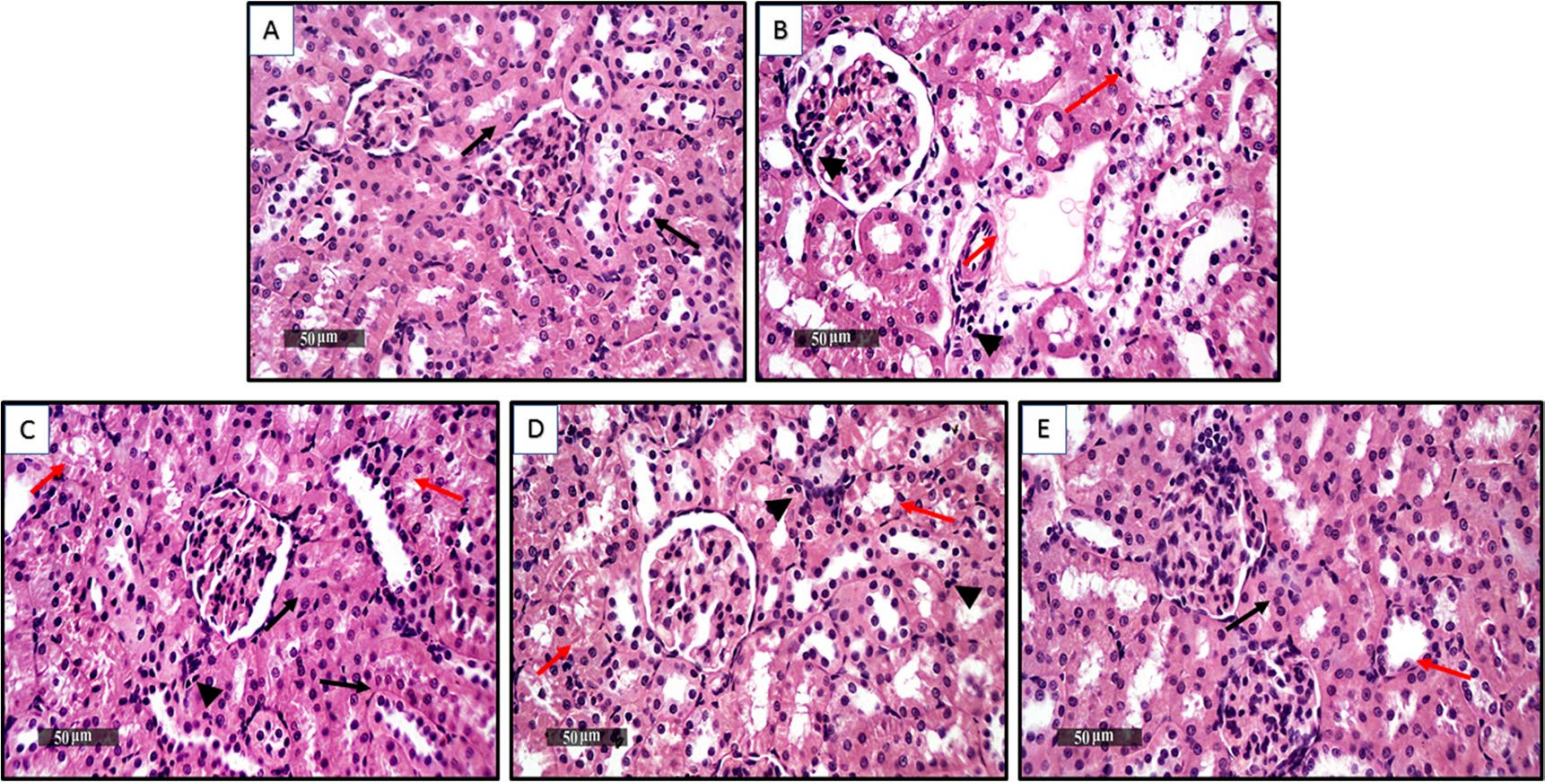
Effect of ORES, DAPA, and Vit. E administration on kidney tissue histological results in rats administered DOX Kidney segments stained with H & E X 400 in photomicrographs. (**A**) Normal control samples, intact tubular epithelium (black arrow). (**B**) DOX-administered rats, cytoplasmic vacuolization (red arrow), interstitial mononuclear inflammatory cell infiltrate (arrow head). (**C**) DOX + ORES-treated rats, intact tubular segments (black arrow), tubular degenerative changes (red arrow), inflammatory cell infiltrates (arrow head). (**D**) DOX + DAPA-treated rats, tubular degenerative changes (red arrow), inflammatory cell infiltrates (arrow head). (**E**) DOX + Vit. E-treated rats, intact tubular segments (black arrow), sporadic occasional degenerative changes (red arrow).

Staining of the kidney sections with H&E was utilized to carry out the histological examination of the renal tissues. Renal specimens from the normal group were examined under a microscope and showed virtually excellent, well-organized renal parenchymal morphological characteristics **(**Fig. 4(A–E)). There was also abundant evidence of seemingly intact renal tubular segments and a nearly intact tubular epithelium. The kidney portions of the DOX-injected group showed notable alterations in the renal architecture. The glomeruli had vascular congestion, invasion of mononuclear inflammatory cells, tubular degradation, and numerous cytoplasmic vacuolizations. However, the level of preventive effectiveness was higher in rats given ORES treatment, as evidenced by the renal parenchyma’s better organized histological characteristics and high proportion of apparent intact tubular segments. In certain tissue slices, minor interstitial mononuclear inflammatory cell infiltrates were observed, along with minimal records of tubular degenerative alterations. Furthermore, moderate protective efficacy of DAPA pretreatment was observed on renal tubular epithelium, with low records of inflammatory cell infiltrates and sustained modest records of tubular degenerative alterations. Last but not least, rats given Vit. E had the greatest preventive efficiency, exhibiting nearly identical records to the normal control samples, including numerous instances of apparent intact tubular segments and sporadic, infrequent degenerative alterations.

### The level of HO-1 immunohistochemical staining is modulated by treatment with ORES, DAPA, and Vit. E

**Fig. 5:**
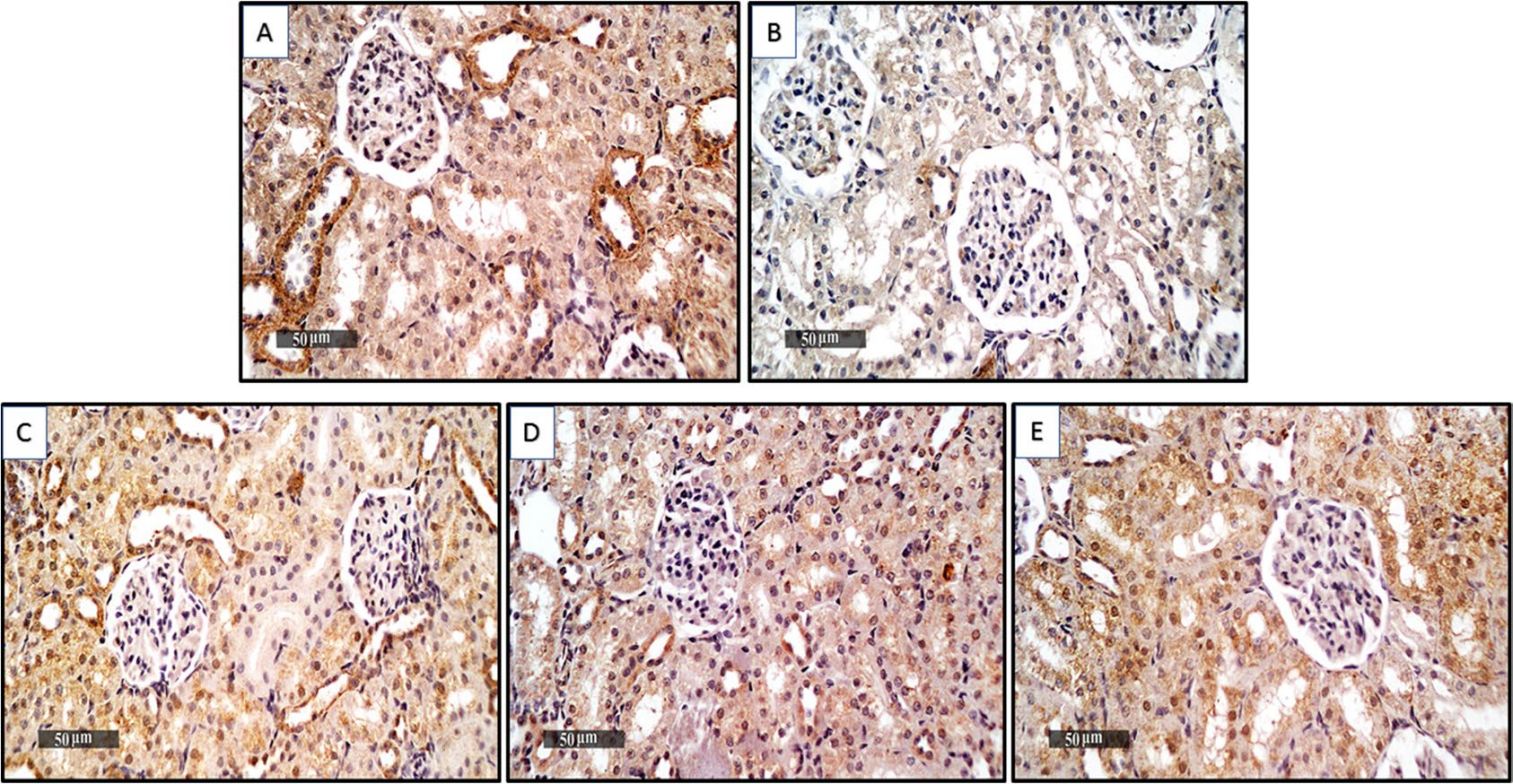
Effect of ORES, DAPA, and Vit. E treatment on HO-1 immunohistochemistry staining level in rats given DOX The DOX sections demonstrated a significant decrease in the expression level of HO-1 protein (Figure 5(B)), while the DOX + ORES, DOX + DAPA, and DOX + Vit. E sections demonstrated a significant increase in the expression levels of HO-1 (Figure 5(C, D, and E)). The normal control section displayed the expression level of prevalent HO-1 protein (Figure 5 (A))

**Fig. 6:**
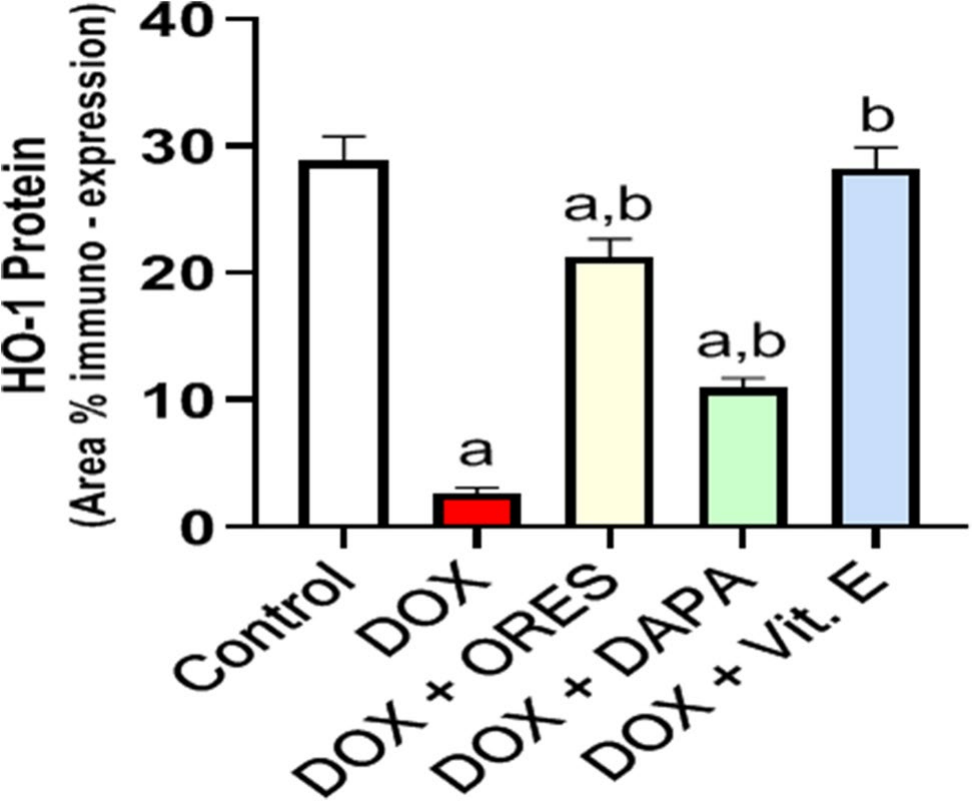
Effect of treatment with ORES, DAPA, and Vit. E on quantitative immune-expression analysis of HO-1 protein level in DOX-administered rats A graph of the quantitative immune-expression level analysis shows the HO-1 protein statistical analysis. The data were displayed as means ± SEM. Using one-way ANOVA and Tukey's test for multiple comparisons across groups at *p* < 0.05, the results were shown to be: ^a^ Significantly different from the control saline group; ^b^ Significantly different from the DOX-administered group

When HO-1 protein expression was assessed by immunohistochemistry, the DOX-treated rats’ results were significantly lower than those of the untreated control group by −0.9-fold **(**Fig. 5(A–E) and Fig. 6). However, co-administration of ORES, DAPA, and Vit. E considerably reduced these alterations compared to the group that received DOX therapy alone, resulting in a notably larger rise in the expression of HO-1 by 7, 3, and 10-fold, respectively.

## Discussion

While the acute nephrotoxicity caused by DOX has been previously examined in various studies, as previously mentioned, no prior research has examined the impact of ORES and/or DAPA on acute nephrotoxicity caused by a single dose of DOX or estimated the full contribution of the PPAR-γ/Nrf-2/HO-1, NF-κB/TNF-α/Keap-1, and Bcl-2/caspase-3/ATG-5 signaling pathways to their nephroprotective actions.

The current investigation proved that ORES and/or DAPA both successfully can ameliorate the acute renal damage induced in rats by a single intravenous dose of DOX, which can be manifested by a decrease in blood levels of creatinine, uric acid, and urea, as well as kidney weight and the kidney/bodyweight. They also increased final body weight and albumin levels in the serum. Reduced kidney caspase-3 concentration was accompanied by decreased serum levels of TNF-α and IL-6. Furthermore, there was an elevation of the Bcl-2 gene, Nrf-2, PPAR-γ, and HO-1 proteins and a downregulation of the renal gene expression of ATG-5, Keap-1, and NF-κB. These results were supported by the decrease in oxidative stress markers and histological alterations.

The propagation of cancerous tumors is now a worldwide phenomenon, and to treat these tumors, doctors tend to utilize chemotherapy in addition to surgical and radiological treatment. Unfortunately, although chemotherapy has impressive results in treating tumors, it has many side effects, which in turn affect the general health of a cancer patient (Ward et al. 2014). Among other cancers, solid tumors, hematological malignancies, and breast cancer can all be more successfully treated with the anthracycline antibiotic DOX (Carvalho et al. 2009). The main adverse effects of DOX that restrict its clinical use are cardiotoxicity as well as renal impairment (El-Sayed et al. 2017a; Songbo et al. 2019). Thus, the purpose of the current investigation was to assess any potential defenses against DOX-induced nephrotoxicity offered by ORES and/or DAPA, utilizing Vit. E as a reference standard. In particular, a study investigated the role of PPAR-γ/Nrf-2/HO-1, NF-κB/TNF-α/Keap-1, and Bcl-2/Caspase-3/ATG-5 signaling networks in the renal injury induced by DOX.

Contrary to the healthy control group, our results indicated that rats given only one intravenous dose of DOX (10 mg/kg) via the tail vein experienced immediate renal injury. Elevated blood levels of urea, uric acid, creatinine, kidney weight, and kidney-to-body weight ratio, as well as a decline in albumin level and final body weight, were the characteristics of this acute renal injury. These conclusions were reinforced by renal histology, which revealed degenerative changes and renal tubule inflammatory cell invasion. The outcomes align with the earlier research (Lahoti et al. 2012; Khames et al. 2017; El-Sayed et al. 2017a). Compared to the rats treated with DOX, the rats given oral doses of 80 mg/kg ORES and 10 mg/kg DAPA over a continuous 14-day period markedly slowed the decline in the kidney function biomarkers and were associated with a notable improvement in the renal histology. Previous studies demonstrating the potent nephroprotective effects of ORES and/or DAPA corroborated these findings (Chen et al. 2011; Abdel-Wahab et al. 2018).

As previously noted, the main way that DOX causes kidney damage is by triggering the oxidative stress cascade, which causes the natural antioxidant defense systems to be inhibited and ROS to be produced (Songbo et al. 2019; Mahzari et al. 2021). This aligns with our findings, which demonstrated that endogenous antioxidant properties were markedly diminished after the administration of DOX. This was evidenced by a decrease in tissue contents of GSH and SOD enzymatic activity, as well as a serum level of TAC and a notable elevation in renal tissues’ MDA and NO_2_^−^ levels compared to normal rats. As an alternative, the simultaneous administration of ORES and/or DAPA with DOX effectively reduces the oxidative damage induced by DOX, as demonstrated by a marked reduction in renal MDA and NO_2_^−^ contents and a notable increase in TAC, GSH, and SOD activity. This promotes cell survival and maintains a robust natural antioxidant system. Our results are consistent with Lorenz et al. (2003), who explained that ORES has powerful antioxidant activity against ROS, and Yaribeygi et al. (2019), who found that, in addition to their capacity to lower glucose levels, SGLT-2 inhibitors like DAPA also possess potent antioxidant qualities that can protect tissues from oxidative harm by lowering the generation of free radicals.

Moreover, oxidative stress generated by DOX promotes a variety of growth and transcription factors involved in the inflammatory pathways (Zhang et al. 2017). Polypeptides, known as cytokines, including IL-6 and TNF-α, control a wide range of critical biological functions, act as immunological and inflammatory mediators (Dinarello 2000), and are linked to glomerular and tubular injury and healing (Sun and Kanwar 2015). The protein called NF-κB contributes to inflammatory pathways by aggravating the inflammatory response through the release of IL-6 and TNF-α. Additionally, it promotes tumor survival by activating Bcl-2, an anti-apoptotic gene (Hayden and Ghosh 2011).

Consistent with other studies (Guo et al. 2013), the present investigation showed that, relative to the control group, DOX significantly raised the renal gene expression of NF-κB, as well as blood levels of IL-6 and TNF-α. Alternatively, rats pretreated with ORES and/or DAPA had significantly lower serum IL-6 and TNF-α levels, and the expression of the NF-κB gene was downregulated. This decline might be explained by the activity of ORES and/or DAPA against oxidative damage and inflammation (Likhitwitayawuid 2021; Hazem et al. 2022).

Furthermore, the protective function of Nrf-2 and PPAR-γ on cells has been demonstrated through their ability to decrease oxidative stress and modulate the inflammatory process (Hsu et al. 2014). The primary negative regulator of Nrf-2 is Keap-1, which leads to the ubiquitylation and degradation of Nrf-2 (Basak et al. 2017). PPAR-γ’s ability to boost the transcription of genes exhibiting antioxidant activity, including glutathione peroxidase, catalase, SOD, and HO-1, accounts for its antioxidant action. Furthermore, in several pathological circumstances, PPAR-γ can reduce the synthesis of factors that promote inflammation, primarily NF-κB (Muzio et al. 2021).

The findings of this investigation demonstrated that activating the Keap-1/Nrf-2 signal pathway inhibits DOX-prompt NF-κB stimulation and inflammatory cytokine production. These results are consistent with Zhang et al.‘s (2021) findings, which demonstrated that administering DOX to animals resulted in a substantial rise in Keap-1 renal gene expression and a considerable drop in HO-1, Nrf-2, and PPAR-γ protein expression. Alternatively, co-treatment of animals with ORES and/or DAPA plus DOX produced a significant reduction in Keap-1 renal gene expression and a marked elevation in HO-1, Nrf-2, and PPAR-γ protein expression.

Multicellular organisms depend on programmed cell death (PCD) for proper development, tissue homeostasis, and structural integrity. PCD encompasses both caspase-dependent apoptosis and non-apoptotic cell death mechanisms, such as autophagy, which is caspase-independent (Jan 2019). Our data corresponds with that of Jan and Chaudhry (2019), who reported that chemotherapy is one of the physical agents that activate cell death. Our results demonstrated a significant decrease in the expression of the Bcl-2 renal gene in conjunction with a notable rise in the tissue caspase-3 and renal gene ATG-5 expression after DOX administration compared to normal animals. On the contrary, in DOX-treated rats, we observed a notable elevation in Bcl-2 renal gene expression and a decrease in caspase-3 and ATG-5 renal gene expression after ORES and/or DAPA pretreatment. These outcomes are consistent with earlier research (Jia et al. 2018; Deger et al. 2022; Yu et al. 2022) that showed how ORES and/or DAPA can suppress autophagy and apoptosis by altering the amounts of apoptotic, antiapoptotic, and autophagic proteins.

## Conclusion

Our research has revealed that DOX treatment can result in apoptosis, autophagy, inflammation, and oxidative damage, all of which can lead to kidney injury. Based on current study findings, DOX-induced acute nephrotoxicity may be prevented by administration of ORES and/or DAPA through changes to the PPAR-γ/Nrf-2/HO-1, NF-κB/TNF-α/Keap-1, and Bcl-2/Caspase-3/ATG-5 pathways. However, further study is needed before a firm statement regarding the potential efficacy of co-administration of DAPA and/or ORES with DOX therapy can be made. This covers both clinical trials and investigations on long-term DOX-induced nephrotoxicity using a variety of animal model species.

## Data Availability

All source data for this work (or generated in this study) are available upon reasonable request.
